# Delivery app workers: working conditions and resistance to the control of digital platforms

**DOI:** 10.47626/1679-4435-2025-1493

**Published:** 2025-09-22

**Authors:** Carolina Cozatti de Camargo, Sergio Roberto de Lucca

**Affiliations:** 1 Faculdade de Ciências Médicas, Universidade Estadual de Campinas, Campinas, SP, Brazil

**Keywords:** information technology, job insecurity, motorcyclists, data management., tecnologias de informação, precarização do trabalho, motociclistas, gerenciamento de dados.

## Abstract

**Introduction:**

The article addresses the work mediated by digital platforms from the narratives of delivery deliverers in Campinas-São Paulo, Brazil.

**Objectives:**

The objective of the study was the influence of algorithmic management on the activities of these workers, and the strategies of resistance to increasing exploitation.

**Methods:**

Qualitative research. 100 platform riders were interviewed between January and July 2023 in three waiting hubs in three shopping centers of the city.

**Results:**

The narratives identified the work process of algorithmic management and undignified working conditions as main thematic matrices and, respectively, the subcategories length of working hours and lack of transparency in the evaluation criteria and remuneration; discrimination and prejudice, and insecurity and work accidents. It was evidenced the power of hubs as a point of exchange of experiences and resistance to the algorithmic manager of platforms.

**Conclusions:**

Despite the discouragement about the future, expressed by most of the deliverers, there is hope in the struggle for the right to decent work and social protection.

## Abstract (pt)

**Introdução:**

O artigo aborda o trabalho mediado pelas plataformas digitais a partir das narrativas dos entregadores de *delivery* em Campinas, São Paulo.

**Objetivos:**

O objetivo do estudo foi investigar a influência do gerenciamento algorítmico sobre as atividades desses trabalhadores e as estratégias de resistência à crescente exploração.

**Métodos:**

Pesquisa qualitativa. Foram entrevistados, entre janeiro e julho de 2023, 100 motofretistas de plataformas em três *hubs* de espera de três shopping centers do município.

**Resultados:**

As narrativas identificaram o processo de trabalho de gestão algorítmica e a precarização das condições de trabalho como principais matrizes temáticas. Respectivamente, as subcategorias de análise foram descritas por meio da extensão das jornadas de trabalho e da falta de transparência nos critérios de avaliação e remuneração, além da discriminação, do preconceito, da insegurança e dos acidentes de trabalho. Evidenciou-se a potência dos *hubs* enquanto pontos de troca de experiências e de resistência ao gestor algorítmico das plataformas.

**Conclusões:**

Apesar do desalento sobre o futuro expresso pela maioria dos entregadores, há esperança na luta por direitos ao trabalho digno e com proteção social.

## INTRODUCTION

The transformations in capitalism since the 1980s have deeply affected both work and the labor force. Globalization and the growth of new technologies reshaped the international division of labor and the forms of work mediated by digital platforms, which are marked by informality, flexible contracts, and the loss of rights and social protection - factors that have increased workers’ vulnerability.^1^

The spread of technology fostered telework, app-based work, and the emergence of a new category of low-paid workers, representing the new service proletariat and the “slaves” of the digital age - the so-called “infoproletariat.”^2^ In a neoliberal context of global structural unemployment, data from the Brazilian Institute of Geography and Statistics (Instituto Brasileiro de Geografia e Estatística, IBGE) indicated that, in 2019, 41.1% of the employed population worked without a formal contract or on a self-employed basis.^3^

Most of this group lives under precarious conditions, as they are citizens deprived of decent, dignified employment.^4,5^ In addition, the COVID-19 pandemic accelerated the pervasive use of these technologies, increasing the overlap between people’s private and public spheres and creating instantaneous, paradoxical work demands.^6^

In this context, work mediated by digital platform companies emerged as an opportunity for “entrepreneurship,” autonomy, and flexibility. However, this type of work - paid strictly according to execution and controlled by algorithmic virtual management - embodies the very essence of exploitation and profit for these platforms.^7,8^ The “platformization” of work has become consolidated worldwide as a new form of labor exploitation, poorly regulated, low-paid, and deprived of rights and social protection.^9^ In Brazil, about 1.5 million people performed services through digital platforms, while 628,000 used e-commerce platforms.^10^

Despite some advances in the regulation of this type of work, our hypothesis is that labor dominated by algorithmic logistics is the main factor contributing to the deterioration of living and working conditions, due to extended working hours and increased exposure to occupational accidents during delivery runs. Some workers argue that overcoming algorithmic management requires collective struggle for better working conditions. Some authors suggest that the survival of digital platform workers could be improved through platform cooperativism.^11,12^

This study aimed to investigate the working conditions of app-based delivery workers and how they experience and resist the precariousness imposed by this new form of algorithmic management.

## METHODS

This qualitative study interviewed 100 app-based delivery workers in Campinas, São Paulo, between January and July 2023. The first “seed” case was identified using the “snowball” technique, starting from a waiting point for calls, known as a “hub,” which led to the indication of other colleagues from 2 additional hubs and their contact networks. This strategy enabled a more diverse sample of motorcycle couriers working through delivery apps.

The interviews were conducted at the hubs, audio-recorded, and followed a semi-structured script with questions on participants’ living and working conditions. Each interview lasted 8-15 minutes. Nonverbal communication was recorded in a field notebook.

Interviews were transcribed and carefully analyzed using discourse analysis,^13^ performed after repeated readings of the transcripts. This analysis consisted of identifying thematic matrices and systematizing content into categories and subcategories to deepen the exploration of the central themes. This approach provided a more comprehensive understanding of working conditions and exploitation dynamics to which delivery workers are subjected. After transcription and floating reading of participants’ narratives, the thematic matrix and 5 categories of analysis were identified.

All participants from the hubs of the 3 largest shopping centers in Campinas volunteered for the interviews; however, 5 of them did not sign the informed consent form and were not interviewed. The recorded interview data were examined in depth through discourse analysis,^13^ systematized and categorized based on the workers’ narratives.

This study was approved by the Research Ethics Committee of the Universidade Estadual de Campinas (Unicamp) under opinion no. CAAE: 61362322.9.0000.5404, on September 8, 2022.

## RESULTS

Almost all participants were male (99%), predominantly aged 26-35 years (46%) and 18-25 years (27%). Respondents self-identified as White (44%), Brown (41%), and Black (15%). Most (77%) had completed high school.

Regarding income from delivery work, most respondents reported daily earnings. Monthly income varied as follows: R$ 1,000-2,000 (13%), R$ 2,000-3,000 (26%), R$ 3,000-4,000 (23%), and more than R$ 4,000 (38%). With respect to occupational accidents, 77% of them reported having experienced at least 1 work-related accident, and among these, 57.27% had been involved in more than 1 incident.

During the interviews, the environmental conditions of the hubs were observed, as well as the informal organization of delivery workers to support colleagues in emergencies, the exchange of experiences in the search for alternative ways to increase earnings, and the strategies of resistance and resilience used to circumvent the traps and injustices imposed by the platforms.

Of the three hubs, two were located in uncovered parking areas, while the third was situated in a covered area near trash bins. There were no restrooms or drinking fountains available, nor a proper place to eat. While waiting for deliveries, participants did not log off the app to avoid penalties and usually ate snacks or cookies, with only a few bringing packed meals for personal consumption. On some occasions, interviews were interrupted due to interference from mall security. The analytical categories from participants’ narratives are presented in Table 1.

**Table 1:** Thematic matrix on the precariousness of platform-based work and categories and subcategories of analysis, Campinas, São Paulo, 2023

Thematic categories	Subcategories of analysis	Key excerpts from narratives
Algorithmic management of work processes	Long working hours	“I’m at level 3. I have 10 minutes to make the delivery. If I’m late, I get punished, and if I’m late too many times because of rain, traffic, whatever, my score drops and I get fewer chances to receive an order. So we’re always racing against the clock.” “If you’re at level 1, you’ll stay logged in the whole day and won’t even make 50 bucks.”
	Lack of transparency in evaluation and pay criteria	“It’s not the same demand every day. Some days you make less, other days you make more. Not knowing how much you’ll earn is a real problem.”
Precarious working conditions	Discrimination and prejudice	“The doorman said he didn’t even like to see a delivery guy. So I’m good enough to bring his food, but not good enough to be seen? Then another client asks me to leave the delivery on the floor. So he’ll pick it up from the floor, but not take it from my hand?”
	Insecurity and work accidents	“I feel like trash. Am I not like everyone else just because I’m a delivery guy?”
		“I’ve had 3 accidents already... most of the time it happens because of the exhaustion and rushing to grab another order.”
		“The problem is all those endless hours working and the risks you face with accidents, cold, and rain.”
		“I’ve had 4 accidents. The last one left my arm paralyzed, and after 3 surgeries, I still can’t stretch it.”
Future of work	Discouragement and disillusion	“I don’t want to live this life anymore. I’m just doing it to survive.”

## DISCUSSION

The profile of the participants in this study is similar to that reported in other research, which highlights the predominance of Black and Brown workers among delivery couriers as well as situations of discrimination and segregation by commercial establishments and clients.^10,14^

Within the analytical categories of the thematic axis, the algorithmic management of work processes was included, as it lies at the core of platform companies’ control, power, and profit. It became evident that the lack of flexibility and autonomy is paradoxical,^6^ since the algorithm ranks and prioritizes orders based on delivery history, imposing long hours of availability so that workers can meet the individually stipulated daily earnings targets needed for survival. In other words, the payment per delivery is variable and dynamic, depending on the number of orders, the availability of couriers at the time, and the individual ranking level set by the algorithm for order prioritization. As a result, in order to secure a minimum daily income, extended working hours are often required.

Delivery workers do not retain the full amount earned from deliveries, as a portion of their earnings is directed to the remuneration of the digital platform.^15^ According to the analyzed reports, this fee represents a significant amount, making it one of the main sources of dissatisfaction among workers. Furthermore, in deliveries to nearby locations or condominiums, even when 3 deliveries are made, the worker is paid for only 1, although the platform charges each client a delivery fee.

For 48% of respondents, some positive aspects of app-based work include flexibility and autonomy in setting their own work schedule and “*not having a boss*.” Additionally, 19% reported greater freedom “*to work whenever I want*.” However, for these so-called “*entrepreneurs*,” most of whom are “*self-employed*,” flexibility and autonomy do not fully materialize, since workers remain bound to the logic of subordinate algorithmic self-management.^16,17^

The core of algorithmic management lies in the triangulation among service providers, clients, and platforms, designed for surveillance and control of both users and workers.^18^ Its programmed adaptability results from the datafication of an enormous volume of information from both users and providers or clients.^19^ Moreover, in algorithmic management of work, the ranking system applied to couriers bypasses the very principles of what would constitute “fair” competition among them. This differentiation between the number of tasks completed and the corresponding payments serves the purpose of controlling and dividing the workforce, while also increasing delivery speed.^20^

The algorithm also determines the time within which tasks must be completed, the payment, and the delivery time, while exerting pressure on workers to accept all orders under the threat of temporary suspension or demotion. At the same time, it implements reward mechanisms through gamification, encouraging service providers to remain logged in longer before returning home. However, greater engagement, expressed through gamified incentives to extend working hours, does not necessarily translate into higher income for workers.^20^

Despite the relevance of this group of workers to consumer society - considered an essential service during the COVID-19 pandemic, when most providers continued working 6-7 days per week, averaging 10 hours a day - their labor relations are increasingly individualized and rendered invisible, leading them to feel socially invisible.^21^

This apparent invisibility is accompanied by exclusion and discrimination. Reports of racial discrimination experienced by delivery workers in their interactions with customers and delivery services, which prevent them from using basic facilities and rest areas, exemplify this marginalization.

Discrimination reflects structural racism. Being predominantly Black and Brown, these workers are embedded in the production and reproduction of inequalities and social injustices that structure Brazilian society. To acknowledge their subordination, regulate and protect workers’ lives, and reduce these inequalities means ensuring the integrity of citizens - especially Black workers - against being relegated to the status of second-class citizens.^14^

In addition to social exclusion, feelings of worthlessness, and precarious working conditions in waiting hubs, workers also face food insecurity and intrinsic factors of the work process that make them captives of platform algorithms. Exhausting workdays contribute to the high incidence of occupational accidents in this group.^22^ Moreover, the work process based on the number of runs compels workers to speed up deliveries in a rush to complete the next one, thereby increasing the risk of accidents.

In this study, 77% of respondents reported having experienced occupational accidents. Among them, 27% had been involved in 2 accidents, while the rest reported more than 3 occurrences. Severe accidents, resulting in more than 30 days of leave, accounted for 18% of cases. In addition to the weight of the delivery bags, poor road conditions and adverse weather increased the likelihood of work-related accidents.

The labor relationship shaped by a logic of “forced entrepreneurship” places the burden of costs and risks on delivery workers under this subordinated management. In the absence of social protection and workplace accident insurance, long-term leaves must be compensated through the solidarity of coworkers. The responsibility for assistance and treatment of such accidents should lie with society and be funded by the Brazilian Unified Health System (SUS), rather than falling on platform companies.

The informality of work in digital platforms places workers in a scenario of neglect, instability, and lack of social protection and prospects for the future. For 71% of respondents, the future is discouraging, 14% considered it uncertain, 12% believed in a positive future, and 3% were unable to answer. Based on the narratives, it became clear that respondents intend to work with app-based delivery only for a short period. Although they recognize there is no future in this type of work, the need for employment to survive leaves them with no way out.

Some limitations of this study should be noted regarding the convenience sample, which showed a predominance of male motorcycle couriers - an aspect that may represent a potential bias. The original design of the field research aimed to reach saturation of the study population. However, as interviews progressed in the hubs, the sample was expanded since most workers showed interest in participating in the study.

Due to interruptions and interference from mall security, hubs were not the ideal setting for conducting the interviews. Many of the motorcycle couriers expressed concern about incoming calls from the apps, which prevented them from providing more detailed responses. Therefore, the context and working conditions at the hubs may have affected the interview content.

## CONCLUSIONS

In a global neoliberal context, where most workers are informal and lack rights and social protection, digital platform work has become the main source of income for delivery workers. In this study, 54% of respondents reported never having had a formal job.

Most participants expressed discouragement about the future, remain captive to algorithmic management, work long hours, experience discrimination, feel unfairly treated due to variable pay, and report that time pressure for each run increases the risk of occupational accidents.

Amid narratives filled with frustration, complaints, and indignation, we observed that waiting hubs serve as collective and strategic spaces that foster the exchange of experiences, articulate common demands, strengthen bonds of solidarity, and contribute to building a collective identity of resistance and political struggle.

Although the demand for formal employment regulation is not a hegemonic banner within this category, we consider the pursuit of the right to dignified work with social protection to be of fundamental importance.
